# Devotion to Family: Coping Strategy to Reduce Burden of Caregiving Stroke Patients

**DOI:** 10.1155/oti/5517436

**Published:** 2025-09-12

**Authors:** Khomarun, Achmad Arman Subijanto, Ninik Nurhidayah, Ari Probandari

**Affiliations:** ^1^Development Extension/Community Empowerment, School of Postgraduate Studies, University Sebelas Maret, Surakarta, Indonesia; ^2^Department of Occupational Therapy, Polytechnics of Health Surakarta, Surakarta, Indonesia; ^3^Department of Public Health, Faculty of Medicine, University Sebelas Maret, Surakarta, Indonesia

**Keywords:** caregiver, coping strategy, devotion, stroke

## Abstract

**Background:** Informal caregivers have played a significant role since stroke survivors were discharged from the hospital. Caregivers carry out stroke survivors' daily activities, which can be burdensome. Caregiver coping strategies to reduce this burden have not been widely studied, especially in Indonesia.

**Purpose:** This study was aimed at exploring the act of devotion as a coping strategy to reduce the burden of caring for stroke survivors.

**Method:** This was an exploratory qualitative study of 30 informal caregivers of stroke survivors who live in a community in Surakarta, selected using purposeful sampling. In-depth interviews to explore the act of devoting as a coping strategy and observations of caregivers were conducted from June 2021 to December 2022.

**Finding:** The data analysis revealed two primary themes: “family ties” and “physical.” The family ties theme emerged with five subthemes: pride, sincerity, resigned, destiny, and responsibility. The physical theme consisted of two subthemes: ability and spirit.

**Conclusion:** Devotion as a coping strategy for stroke informal caregivers in the community plays a crucial role in maintaining the continuity of care. It involves pride, sincerity, resigned, destiny, responsibility, ability, and spirit. Further research on the results of these findings is needed on how caregivers maintain their quality of life as they begin to become caregivers.

## 1. Introduction

There are more than 12.2 million new stroke cases worldwide each year. The World Stroke Organization (WSO) Global Stroke Fact Sheet 2022 states that stroke remains the second leading cause of death and the third leading cause of disability worldwide [1]. Stroke affects about 795,000 people annually in the United States and is the fifth most common cause of death and disability [2]. In Asian countries, the prevalence of stroke in Indonesia is 293.33 per 100,000 people, much higher compared to Thailand (185) and Korea (159) but still lower than China (719) and India (545) [3].s

Stroke survivors' health is greatly impacted by the condition of their brain [4]. Cognitive problems affect a lot of survivors [5]. Survivors may have speech and memory issues [6], feel as though every element of who they are has been compromised [7], and have physical, emotional, and social limitations that limit their ability to engage in everyday tasks like eating, moving, and taking a shower. Musculoskeletal, sensory, and medical issues are additional sources of disability in stroke survivors [8]. Another study in the Himalayas for patients 3 months after stroke showed that 61% of patients experienced depression and disability, which were the main risk factors for low quality of life in patients [9]. Stroke-related disability was measured using the Modified Rankin Scale (31%); the London Handicap Scale (2.5%); the Barthel Index (12.5%); the World Health Organization's International Classification of Functioning, Disability, and Health (19%); Katz's Index of Independence in Activities of Daily Living (19%), and Functional Independence Measurement (6.25%) [10].

Informal caregivers are usually friends, family, or other acquaintances [11–13]. They may find it difficult to provide stroke survivors with long-term care [14]. A pilot study conducted by Kumar et al. [15] on 22 caregivers of stroke patients in India found that the most important things for caregivers were health information provided by healthcare professionals and the need for social support. Furthermore, financial and relationship issues were also a concern. To address these issues, the most common strategy used was acceptance of the situation. The caregiver burden has detrimental effects on quality of life, care delivery, and physical and mental health [16]. To mitigate these effects, retain the distance and avoid taking on more responsibilities [17]. But there are also good deeds, like dedicating one's life to giving stroke victims gentle, loving care without seeing it as a burden [13]. Disagreements with other carers may have social, economic, psychological, or bodily repercussions. Even though the issues are extremely complicated, each person has coping mechanisms to lessen and even completely eradicate them [15, 18].

Many studies on the experience of providing care for patients who have had a stroke, including the coping mechanisms employed, are currently available. There has not been research done on using devotion as a coping mechanism in literature, especially for Indonesian stroke survivors' carers. To better understand devotion as a coping mechanism for Indonesian poststroke caregivers, this study will be conducted.

## 2. Method

This research applied a qualitative research design with an exploratory phenomenological approach to investigate individual caregivers' perspectives on how to cope as coping strategies during their time as caregivers. The research protocol received ethical approval from the Medical and Health Research Ethics Committee RSUD Dr. Moewardi, Surakarta, Indonesia.

### 2.1. Participants

Purposive sampling was performed to contact 35 eligible participants; five could not make it to the interview due to unwillingness to participate. Participant inclusion criteria were (1) caring for a patient for at least 6 h a day, (2) having cared for a patient for at least 6 months, (3) having a family relationship with the patient, (4) not being paid as a caregiver, and (5) not working as a health worker. Thus, a total of 30 caregivers were involved in the analysis. Information in this study was disseminated to health workers, village health officers, and community members who visited the Integrated Health Service Centers, known as *Posyandu*, in the community. The first author, helped by enumerators, people from the areas, carried out these activities. People in the community provided information on the names and addresses of individuals in their area who have been hospitalized due to stroke. Using this information, the first author and/or enumerators visited the homes of stroke caregivers when they met the inclusion criteria. The participants explained comprehensive information about the study procedure and protocol and signed an informed consent form if they agreed to participate in the research. It was also explained that the researcher would visit several times if the information obtained previously was not complete, according to the information needed or deeper digging. Information was collected through face-to-face interviews with caregivers. Various efforts were made so that participants did not feel embarrassed, afraid, and were willing to express their experiences as caregivers openly, including asking questions to caregivers in a relaxed manner like ordinary and open conversations, providing the author's contact number to participants if they wanted to ask or ask for clarification on something, and several times the author or enumerator visited the patient's house even though it was only to ask for news while rechecking the information on the answers that had been given previously. Caregiver participant demographic data was collected at the beginning of the interview, including gender, age, occupation, relationship with the patient poststroke, and length of caregiving (Table 1). Meanwhile, the 2–3 weeks observation was carried out by an observer who was someone other than the caregivers who lived in the same house or were the closest neighbors of the stroke survivor so that they would know what the caregiver does every day. The observation was carried out after the observers had signed the consent form as participants. Without knowing caregivers, observers observed 29 items of daily living activities of stroke survivors helped by caregivers, as described in Table 2. It was also explained that both caregivers and observers could withdraw from the study at any time during the process. During this study, researchers were very open to receiving information from individuals or the community if there were stroke patients around them.

Observation results in Table 2 show that informal caregivers carry out various routine and tiring activities every day. In the morning, the most frequent activities are preparing breakfast, washing the patient's clothes, and accompanying the patient. Similar activities continue throughout the day, with differences in intensity. For example, the afternoon is marked by a high frequency of accompanying the patient for longer periods and preparing dinner. In the evening, the caregiver's focus shifts to looking after and accompanying the patient and being ready to wake up in the middle of the night if needed. In addition to daily routines, informal caregivers also carry out various additional activities such as cleaning the room or house, managing the patient's finances, and taking the patient to health services. This shows that the role of caregivers is not only limited to the patient's physical needs alone but also includes managerial and logistical aspects that are important for the overall recovery of stroke patients. This role is very complex and demands a high level of responsibility, so there needs to be adequate attention and support for informal caregivers.

### 2.2. Data Collection

The preliminary study was conducted through interviews and observations with three caregivers living in a village in the District Boyolali, Province of Central Java, Indonesia, aiming to identify the problems to be studied and develop questions to focus the research topic. The results of the preliminary study observations provide an overview that caregiver actions to help stroke patients do not recognize time from morning to morning. Based on the observations, a checklist was developed to assess the assistance provided by caregivers to stroke patients. Additionally, from the preliminary study interviews, questions were formulated that focused more closely on the research topic. For example, before conducting a preliminary study, the questions prepared were “Try to tell me whether you feel bored or fed up with the activities you do every day now? Why are you bored/not bored? How about your spirit/spirit in being a caregiver now?” After the preliminary study, broader questions arose, including asking “Try to tell me whether being an informal caregiver now has more problems or not (as usual as before)? What are the problems, and why are they problems? Do you often feel hopeless because you are a caregiver? Try to explain why you are hopeless/not hopeless? After becoming a caregiver, have you become someone who gets angry easily for no apparent reason? Explain why/how this happened.”

The initial meeting with the informant to obtain data from participants did not involve directly asking in-depth questions; instead, it began by asking only for data on the informant's demographics. Friendly communication was built gradually with the aim of not causing feelings of hesitation, shame, and covering up the information conveyed. The next meeting began with more general questions about the participants' activities before becoming caregivers, while deeper and more gradual interviews were conducted after several meetings and after the atmosphere had become more fluid. Interviews are conducted when the participant feels comfortable. Face-to-face interviews with caregivers were conducted using open-ended questions which have been prepared. The participants were asked to describe their experiences in relation to caregiving that related to the study. Each interview lasted 45–60 min. During the interview, with the permission of caregivers, information was recorded using a digital voice recorder. Interviews were transcribed and translated into Indonesian. Meanwhile, the observer observes the caregiver about the daily activities he or she carries out to help stroke patients. Observers put a “V” mark on the activity list according to what the caregivers do. Finally, all the information is included in the data analysis.

### 2.3. Preunderstanding

The authors have different backgrounds. The first author is a doctoral student who has an interest in the caregiver area of neurological conditions. The second and fourth authors have a medical background, and their expertise is in the field of public health. The third author is an occupational therapist who has an interest in the field of geriatrics and social rehabilitation.

### 2.4. Data Analysis

All interview data was recorded and transcribed word for word into Indonesian, and then the researcher and enumerator discussed and checked and rechecked to agree on the accuracy of understanding the meaning of the sentences expressed by the participants. Data analysis is carried out using NVivo 14 software to identify ideas that produce categories. Open coding was first carried out with the analysis of interview transcription data. The results of open coding yielded codes for key phrases that were often said by informants. Second, axial coding is carried out, namely, grouping the codes resulting from open coding according to closer categories. The observation results can be described that the experience of informal caregivers while caring for stroke patients shows a major change in their personal lives, which is colored by physical, emotional, and financial pressure. Despite facing various challenges such as boredom, sleep disorders, anxiety, and economic problems, they still show enthusiasm and readiness to continue their caregiving role because of their sense of responsibility, affection, and spiritual and family support.

## 3. Result

Based on the data on the characteristics of informal caregivers of stroke patients, as many as 30 respondents, most of whom are women, with a total of 18 people (60%), while men are 12 people (40%). This shows that the role of informal caregiving is more often carried out by women, which is most likely related to cultural and social values that place women as caregiver figures in the family.

In terms of age, most caregivers are in the age group above 60 years, which is 14 people (46%). Meanwhile, eight people (27%) are in the age group below 50 years and between 50 and 60 years. This indicates that many caregivers are already in their old age, which can increase the burden of caregiving due to physical limitations or health problems that accompany old age.

The relationship between caregivers and stroke survivors is dominated by the wives of the survivors, as many as 17 people (57%). This shows that the responsibility for caring often falls on the partner, especially the wife. In addition, children also take on the role of caregivers (17%), followed by husbands (23%) and older siblings (3%). This difference in roles reaffirms the existence of norms in the family that encourage partners or close family members to be directly involved in caring.

Two main themes were obtained after qualitative analysis of transcribed interviews, namely, “family ties” and “skills.” Family ties consisted of pride, sincerity, resigned, destiny, and responsibility, and skills consisted of ability and spirit, as portrayed in Table 3.

### 3.1. Family Ties

The results of the research illustrate that the act of caring for stroke sufferers has many positive experiences as well as negative sides. Serving is a positive experience for some people because of the emotional attachment and compassion they have for their patients. Family ties include the following.

### 3.2. Proud

A 55-year-old man who helps with daily activities for his sibling who suffered a stroke stated, “Being a caregiver creates a feeling that caring for stroke survivors can foster a sense of pride because survivors are no longer able to carry out their activities due to illness.” Another caregiver said:

Why am I embarrassed? It's because I take care of someone that I feel proud of. So, I am not embarrassed. (caregiver #10, wife, 66 years old)

I am proud, if I take care of sick people, there must be wisdom behind it. The important thing is that I do everything sincerely. (caregiver #12, older brother, 55 years old)

### 3.3. Devoted

Participants described the reason for caring as a form of giving back to their parents. The 26-year-old son said, “Yes, because I feel like there is a responsibility, you know, as a child I have to be filial to my parents no matter what the situation is,” while a 25-year-old daughter stated, “That is natural because my mother. So, I think it's probably a change. Previously, my mother took care of me; now I take care of my mother” (caregiver #16, daughter, 25 years old).

### 3.4. Sincere

Expressions of sincerity came from a number of participants. A 57-year-old wife said, “… because it is my obligation, Sir, everything is from Allah, tested by Allah, yes… accepted with sincerity, patience.” Other participants stated:

Still, keep up the enthusiasm… I have to be sincere, I have to be strong. (caregiver #02, son, 44 years old)

Yes, I feel the maximum, sometimes I feel tired, but just do it sincerely. (caregiver #03, daughter, 36 years old)

No, sir, it's normal, because in dealing with me, I have been sincere and patient, maybe you will get used to it. (caregiver #17, wife, 57 years old)

### 3.5. Resigned

Several participants mentioned that everything happened because no one wanted it, so they left everything to God. As mentioned by a husband, 70 years old, “…. I have resigned myself to the fact that at any time something happens” (caregiver #23, husband, 70 years old). Some caregivers also spoke about this resignation. Another man is a husband, 70 years old, said, “Everyone surrenders to the Almighty.”

### 3.6. Destiny

Fate is a decree about good or bad that comes from God so that humans cannot avoid it, including being a caregiver. As stated by a man, aged 26 years, the son of a stroke sufferer, “….No, yes, because it was fate and I did my best, but that was the will above” (caregiver #1, son, 26 years old). The other caregiver's narrative expressions are:

Don't be embarrassed, because this is fate, it's the way it is. (caregiver #03, son, 44 years old)

It's fate, Bro, just do it. (caregiver #18, wife, 65 years old)

I consider this fate from the Almighty. (caregiver #20, husband, 55 years old)

### 3.7. Responsibility

The subtheme of the reason for caring for stroke survivors is due to the responsibilities and duties of family members. One participant, being a wife, 60 years old, who has been caring for her stroke husband for 36 months, said “Do not get bored because of responsibilities as a wife” (caregiver #15, wife, 60 years old). A husband, 67 years old, who has been caring for 72 months his stroke-stricken wife expressed, “Who else should be responsible if not the husband, because his sick wife?”

### 3.8. Ability

No one expects to be a caregiver for a stroke sufferer, so it is normal not to have enough skills to care for them. Not all caregivers are health workers, so their role as caregivers is only forced, and they do not have any skills related to caring for stroke sufferers.

Yes, if there is as little influence as possible, Sis, as much as possible, the important thing is that right now I am taking care of him as much as possible. (caregiver #09, wife, 62 years old)

### 3.9. Spirit

One of the expressions expressed by caregivers is spirit, which is a very strong feeling experienced by caregivers that is able to develop high will power, as in the following expression:

Never give up, just be enthusiastic. If I complain, then I won't be pleased. Even though I'm tired, just keep spirit. (caregiver #02, son, 44 years old)

Yes, I'm bored because every day the work is monotonous, and there is a feeling of boredom. Yes, just encourage yourself. Keep your spirits up to be healthy, to be strong. If we are not enthusiastic, we will feel sorry for the mother too. (caregiver #03, son, 44 years old)

Yes, keep up the spirit. Until whenever we have to be enthusiastic, optimistic. Because of healing, we have tried everywhere to go to doctors, herbs, therapy, but healing is still God's. So, stay optimistic, you will recover. (caregiver #9, wife, 62 years old)

Apart from devotion explained above, the experience of caring for stroke sufferers also creates various problems, including stigma, problem of financial, time, psychological, physical, and social aspects, as explained in Table 4.

Other problems that arise from caring for stroke survivors are expressed by caregivers below.

### 3.10. Self-Stigma

Self-stigma arises when caregivers feel incapable, unworthy, or not good enough to carry out the task of caring for a sick family member, as expressed by the following caregiver: “I'm doing my best that I can to take care of him… but honestly, I can't treat him” (caregiver #12, older brother, 55 years old); “I often thought whether I can do it or not, right or wrong what I done in caring for him, preparing everything for him, sir” (caregiver #17, wife, 57 years old).

### 3.11. Stigma From Sufferers

Apart from self-stigma, interview results also found that stigma was given by sufferers to informal caregivers.

She keeps getting angry, I won't….because sometimes she doesn't believe me… it makes me desperate. (caregiver #24, husband, 67 years old)

… she often gets angry by herself,… throws outbursts by herself… because she feels I don't understand her. (caregiver #28, son, 30 years old)

### 3.12. Financial

Financial problems are also experienced by caregivers due to many changes in their lives.

Before my mother got sick, I could use all my time for business… Now I can't… financial needs are a problem. (caregiver #3, son, 44 years old)

Yes, it's very different. The difference… the economy can't function. (caregiver #28, son, 30 years old)

### 3.13. Rest/Sleep

Caring for a stroke patient causes the caregiver to lack sleep and rest.

Often, yes… I sleep restlessly… I think my husband's illness won't get better…. (caregiver #14, wife, 55 years old)

My sleep is restless… I often stay up too late waiting for my mother to go to bed. (caregiver #16, daughter, 25 years old)

### 3.14. Angry

The caregiver's expression of anger can be described by the following expressions conveyed by the caregiver.

…it's normal, sir, family problems are bound to have their own. I'm tired, angry, and fed up with caring for my husband who's had a stroke. (caregiver #15, wife, 60 years old)

I feel angry, but I don't know who to be angry at. (caregiver #17, wife, 57 years old)

### 3.15. Afraid

Caregivers also feel anxious when they see the condition of stroke sufferers, as they expressed.

I was anxious… I felt afraid… I wondered whether my mother would recover or not. (caregiver #16, daughter, 25 years old)

Another caregiver stated,

Yes, I did… when my husband had seizures and stiffness, I was worried… I was scared. (caregiver #26, wife, 64 years old)

### 3.16. Confused

A 57-year-old wife admitted to being confused while caring for her husband who had a stroke. “I… when I first started caring for my husband… I often cried… I was confused about what I should do” (caregiver #17, wife, 57 years old).

### 3.17. Tired

The feeling of tiredness from caring for a stroke patient was also expressed by the caregiver, a 62-year-old wife: “Yes, perhaps because of age. As I get older, caring for my husband feels easily tiring…” (caregiver #9). Another caregiver stated, “Sometimes, I feel tired caring for my husband who has had a stroke…” (caregiver #27, wife, 54 years old).

### 3.18. No Skill Caregiving

All caregivers are not professional health workers, so the process of learning to care is done independently and through trial and error.

I don't have any skills…. I just practice, it's okay to be a little lacking, as long as I learn every day what makes me comfortable. (caregiver #12, older brother, 55 years old)

I learn on my own, try things out, no one teaches me, if I make a mistake, I fix it. (P19)

### 3.19. Sick

Caregivers often experience overwhelming caregiving, which makes them vulnerable to illness. “Often when I'm caring for my sick wife, there's no one to replace me, so I'm forced to keep looking after the patient, so I often get sick” (caregiver #25, husband, 67 years old).

### 3.20. Gather With Others

Some people said they were unable to or had reduced social activities after having to care for a family member who had a stroke.

I've done so much for the community. Now I just stay at home and take care of my husband. (caregiver #27, wife, 54 years old)

… it doesn't feel good, but what else can I do when I'm taking care of her? People already know. (caregiver #22, husband, 63 years old)

## 4. Discussion

The results of this study indicate that the experience of being an informal caregiver is not just a caregiving activity but rather a long process that touches on the realm of self-identity, social relationships, and individual and family dynamics. However, behind the burdens experienced, informal caregivers show various forms of resilience and empowerment that are worthy of appreciation. Qualitative research on coping strategies to reduce the burden of caring for stroke patients on informal caregivers provides an in-depth view of the coping mechanisms applied. Informal caregivers, often family members, play a key role in caring for stroke patients at home, which carries a physical and emotional burden. This research identified several main themes, including family ties, passion, and devotion as coping strategies. In comparing the results of this study with previous research, several similarities and differences can be found.

### 4.1. Devotion as a Coping Strategy

In this study, caregivers used devotion as the main strategy to overcome the burden of care. This devotion includes feelings of sincerity, surrender to fate, and responsibility towards the family, which encourages caregivers to continue caring for stroke patients even though they face many challenges. A previous study by Amaliyah [19] also found that caregivers involved in the care of stroke patients tend to show high devotion because of their strong emotional bond with the patient, which is often driven by cultural and religious values. For example, in the Indonesian context, cultural values that value family ties are very strong, where children feel obliged to care for their parents when they are sick. This is similar to research by Qiu et al. [20], which shows that caregivers in Chinese culture are also bound by a moral obligation to care for their parents as a form of filial piety. This devotion is a factor that strengthens the caregiver's emotional and mental coping.

### 4.2. Caregiver's Psychological and Physical Burden

Apart from the service aspect, this research also highlights the various burdens faced by caregivers, including psychological burdens, such as anxiety, stress, and depression, as well as physical burdens due to intensive care activities. This finding is supported by a study by Borah et al. [21], which identified that caregivers of stroke patients often experience quite severe mental and physical stress. This stress arises from the ongoing responsibility of caring for patients who require assistance with almost all daily activities. Another study by Kristanti et al. [22], who studied caregivers of cancer patients, found that the physical and psychological burdens were similar, especially because caregivers had to continuously support patients who had physical and cognitive limitations. This burden is often exacerbated by a lack of external support, so caregivers must find their own ways to survive difficult situations.

### 4.3. Family Ties

Strong family ties, as found in this study, are often the main source of strength for caregivers. In this study, family ties emerged in various forms, including a sense of pride in being able to care for a family member and a belief that their role in care was a predestined responsibility. This is in line with the findings of Kazemi et al. [17], which show that caregivers who feel an emotional responsibility toward patients often develop family-centered coping strategies, including seeking emotional support from other family members.

However, it is important to note that although family ties can be a source of strength, they can also be a source of stress for caregivers, especially if there is disagreement between family members regarding how to care for the patient or the division of tasks. In some cases, this disagreement can worsen the psychological burden experienced by the caregiver, as found in the study of Iqbal et al. [18] regarding caregiver experiences during the COVID-19 pandemic.

### 4.4. External Support in Burden Reduction

This research shows that caregivers involved in the care of stroke patients often have to rely on internal resources, such as emotional strength and enthusiasm. However, other studies suggest that external support, such as from health professionals, the community, or social assistance programs, can significantly reduce caregiver burden. An et al. [23] note that community-based interventions can provide practical help, while psychological support can help reduce emotional burden. Meanwhile, in research by Utaisang et al. [24] in Thailand, it was found that caregivers who had access to a strong social network were better able to manage the burden of care. Those who have a supportive community are able to share caregiving duties with other family members or even get outside help.

## 5. Conclusion

Devotion as a coping strategy for caregivers of stroke patients is an important element that appears in various studies. The experiences of informal caregivers while caring for stroke patients show major changes in their lives, both in terms of daily routines, personal activities, and psychological and financial conditions. It is human nature that caregivers face various challenges, such as physical fatigue, sleep disturbances, anxiety, emotional stress, and a fairly heavy financial burden due to the costs of care and daily needs. Nevertheless, most caregivers continue to show enthusiasm and readiness to continue caring for stroke patients, based on a sense of responsibility, sincerity, compassion, and religious motivation. This reflects that the role of an informal caregiver requires strong physical, mental, and financial resilience and support from the social and family environment. This service must also be balanced with adequate psychological and physical support so that caregivers do not experience excessive fatigue. Interventions from external parties, such as community support or health services, can play an important role in reducing the burden experienced by caregivers. Therefore, a multidisciplinary approach involving the support of family, community, and health professionals is needed to improve caregiver well-being. In general, their motivation can be seen from internal factors (beliefs, obligations, and expectations) and external factors (family support, physical health, and patient conditions).

## Figures and Tables

**Table 1:** Caregiver participant data.

**Characteristic**		**Caregiver ( ** **n** = 30** )**
Gender	Man	12 (40%)
	Woman	18 (60%)

Age	< 50 years	8 (27%)
	50–60 years old	8 (27%)
	> 60 years	14 (46%)

Work	Housewife	13 (43%)
	Private employees	5 (17%)
	Businessman	1 (3%)
	Farmer	4 (13%)
	No work	2 (7%)
	Teacher	2 (7%)
	Pension	3 (10%)

Relationship with stroke survivors	Wife	17 (57%)
	Husband	7 (23%)
	Son/daughter	5 (17%)
	Sibling	1 (3%)

Length of caregiving	1–2 years	12 (40%)
	2–3 years	1 (3%)
	> 3 years	17 (57%)

**Table 2:** Items of daily living activities helped by caregivers.

**Morning**	**Afternoon**	**Evening**	**Night**	**Other activities**
1. Wake up	1. Prepare lunch	1. Bathing	1. Look after/accompany the patient	1. Clean the room/house
2. Bathing	2. Feeding	2. Change clothes	2. Waking up in the middle of the night to help the patient sleep, accompany, or something else	2. Manage patient finances
3. Change clothes	3. Look after/accompany the patient	3. Prepare dinner	3. Give medicine	3. Take the patient for examination/treatment to the doctor/therapy
4. Prepare breakfast	4. Give medicine	4. Feeding	4. Other activities to help patients	4. Accompany patients for sports/walking/visiting/others
5. Feeding	5. Other activities to help patients	5. Look after/accompany the patient		
6. Wash clothes		6. Give medicine		
7. Look after/accompany		7. Other activities to help patients		
8. Give medicine				
9. Other activities to help survivors				

**Table 3:** Caregiver's coping strategy.

**Theme**	**Subthemes**	**Category**	**Example of a quote**
Devotion as a coping strategy	Family ties	Proud	Taking care of mother…….should be proud, because it's rare for anyone to take care of their mother, especially when they cannot do anything. (caregiver #3, son, 44 years old)
		Devoted	I feel like there is a responsibility, you know, as a son I must serve my parents and how things are going (caregiver #01, son, 26 years old)
		Sincere	The important thing is that I do everything sincerely, Sis. I have no strings attached (caregiver #12, brother, 55 years old)
		Resigned	I never let go of your prayers, miss. I leave everything to Allah. (caregiver #6, wife, 76 years old) “If my heart is sincere… that is a test from Allah SWT…” (caregiver #20, husband, 55 years old)
		Destiny	Surrendering everything to God, feeling more at peace with a religious approach (caregiver #26, wife, 64 years old)
		Responsibility	“…this person is my husband, life and death are with my husband.” (caregiver #11, wife, 62 years old) “…this is my obligation, a trial for me too.” (caregiver #13, wife, 60 years old) It's normal, no shame because it is fate (caregiver #14, wife, 51 years old) “It does not make me bored, because it's an obligation…accept it sincerely, patiently.” (caregiver #17, wife, 57 years old) Yes, she is my wife, I must be responsible for caring (caregiver #24, husband, 67 years old)
	Ability	As able as possible	I feel like I can take care of it, as best as I can; it's just what the father cannot help with (caregiver #02, wife, 39 years old)
		Spirit	No…… I never get bored. The main enthusiasm… never gets bored (caregiver#2, wife, 39 years old) “That's not called a devout, a devout does not know despair.” (caregiver #9, daughter, 44 years old) “…. I get continuous support from the children, so I am enthusiastic.” (caregiver #22, husband, 66 years old)

**Table 4:** Problems arise in caregivers.

**Category**	**Subcategory**	**Example of quote**
Stigma	Self-stigma	I'm disappointed with myself. I sometimes get angry with myself, do not know why (caregiver #27, wife, 54 years old)
	Stigma from sufferers	Yes, sometimes he feels like I'm not capable enough, because I'm just a layman, not a health professional, plus I'm a man, so that's how it is; sometimes I still cannot be trusted (caregiver #1, son, 26 years old)
Finance	Finance	The need for money may also be a problem, because my child also seems to need money (caregiver #2, son, 44 years old)
Time	Rest/sleep	I cannot sleep at night. If we are both sleeping, she'll call out to pee, it's itchy, she'll call out. I cannot always sleep well. If I'm stuttering, it gives me a headache, dizziness (caregiver #5, son, 31 years old)
Psychology	Anger	Yes, sometimes I get angry when she screams. The reason is that… how is he sometimes… her words are shouting like that (caregiver #5, son, 31 years old)
	Worried	Well, what do you think the main thing is that I feel a little worried (anxious), Sis, the main thing is, I hope she gets well soon, keep praying so that she gets well soon and healthy like that (caregiver #2, son, 44 years old)
	Confused	My body gets goosebumps, family members are as sad as anything if their husband dies, I feel sorry for them, I do not want to. I thought I was going to die, Mom… I was ready to die… try to imagine that (caregiver #5, son, 31 years old)
Physically	Tired	If I do not give up, Sis, it's just that sometimes I get annoyed, sis, because when my body is tired, it's not fit, but there's still work to do at home (caregiver #10, wife, 55 years old)
	No skills caregiving	As much as possible, the important thing right now is that I take care of my husband as much as possible (caregiver #9, wife, 62 years old)
	Sick	My head feels like it's spinning, heavy. My body also just hurts, now I hear talk of wanting to die, and I have a headache (caregiver #5, son, 31 years old)
Sociability	Gather with other people	Yes, I feel bad not to be together, but how else will people take care of her (caregiver #22, husband, 63 years old) Yes… I do not go out anywhere, Sis, I just stay at home watching TV, being a housewife, and taking care of my father (caregiver #8, daughter, 44 years old)

## Data Availability

The data that support the findings of this study are available from the corresponding author upon reasonable request.
